# Supplementary material for: Micrometer-thick and porous nanocomposite coating for electrochemical sensors with exceptional antifouling and electroconducting properties
Source: Nat Commun. 2024 Feb 8;15:711. doi: 10.1038/s41467-024-44822-1 (PMC10853525; doi:10.1038/s41467-024-44822-1)
Supplement: Supplementary file 1 — Supplementary information [file 41467_2024_44822_MOESM1_ESM.pdf]

Supplementary information

Micrometer-thick and porous nanocomposite coating for electrochemical sensors with exceptional antifouling and electroconducting properties

Jeong-Chan Lee[1,2,#] , Su Yeong Kim[2,#], Jayeon Song[3,4,5,#], Hyowon Jang[3], Min Kim[2], Hanul Kim[6], Siyoung Q. Choi[6], Sunjoo Kim[7], Pawan Jolly[1], Taejoon Kang[3,8,*], Steve Park[2,*], Donald E. Ingber[1,9,10,*]

[1] Wyss Institute for Biologically Inspired Engineering, Harvard University, Boston, MA 02115, USA

[2] Department of Materials Science and Engineering, Korea Advanced Institute of Science and Technology (KAIST), Daejeon 34141, Republic of Korea

[3] Bionanotechnology Research Center, Korea Research Institute of Bioscience and Biotechnology (KRIBB), Daejeon 34141, Republic of Korea

[4] Center for Systems Biology, Massachusetts General Hospital Research Institute, Boston, MA 02114, USA

[5] Department of Radiology, Harvard Medical School, Boston, MA 02114, USA.

[6] Department of Chemical and Biomolecular Engineering, KAIST, Daejeon 34141, Republic of Korea

[7] Department of Laboratory Medicine, Gyeongsang National University Hospital, Gyeongsang National University College of Medicine, Jinju-si, Gyeongsangnam-do 52727, Republic of Korea

[8] School of Pharmacy, Sungkyunkwan University (SKKU), Suwon-si, Gyeongi-do 16419, Republic of Korea

[9] Vascular Biology Program and Department of Surgery, Boston Children's Hospital and Harvard Medical School, Boston, MA 02115, USA

[10] Harvard John A. Paulson School of Engineering and Applied Sciences, Harvard University, Boston, MA 02115, USA

*Corresponding authors. Email: don.ingber@wyss.harvard.edu (D.E.I.), stevepark@kaist.ac.kr (S.P.), and kangtaejoon@kribb.re.kr (T.K.)

[#] These authors contributed equally

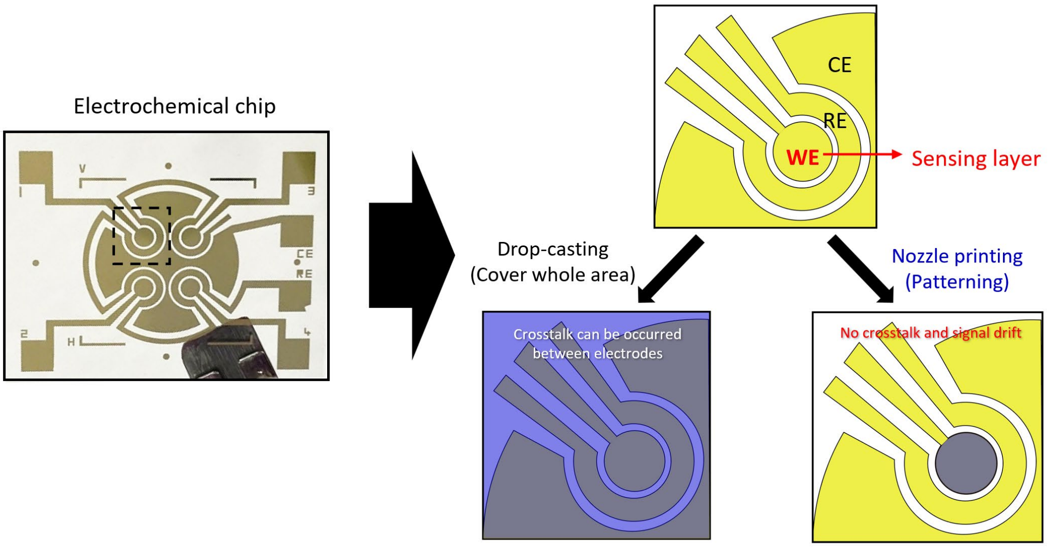

Supplementary Figure 1. Illustration depicting the difference between drop-casting and nozzle printing. Drop-casting, despite its simplicity, leads to coating all electrodes—working, counter, and reference electrodes—with the composite, potentially causing crosstalk due to the presence of nanoelectrodes. On the other hand, nozzle printing selectively applies the composite solely to the working electrode, where target binding events occur, enabling the precise extraction of electrical signals.

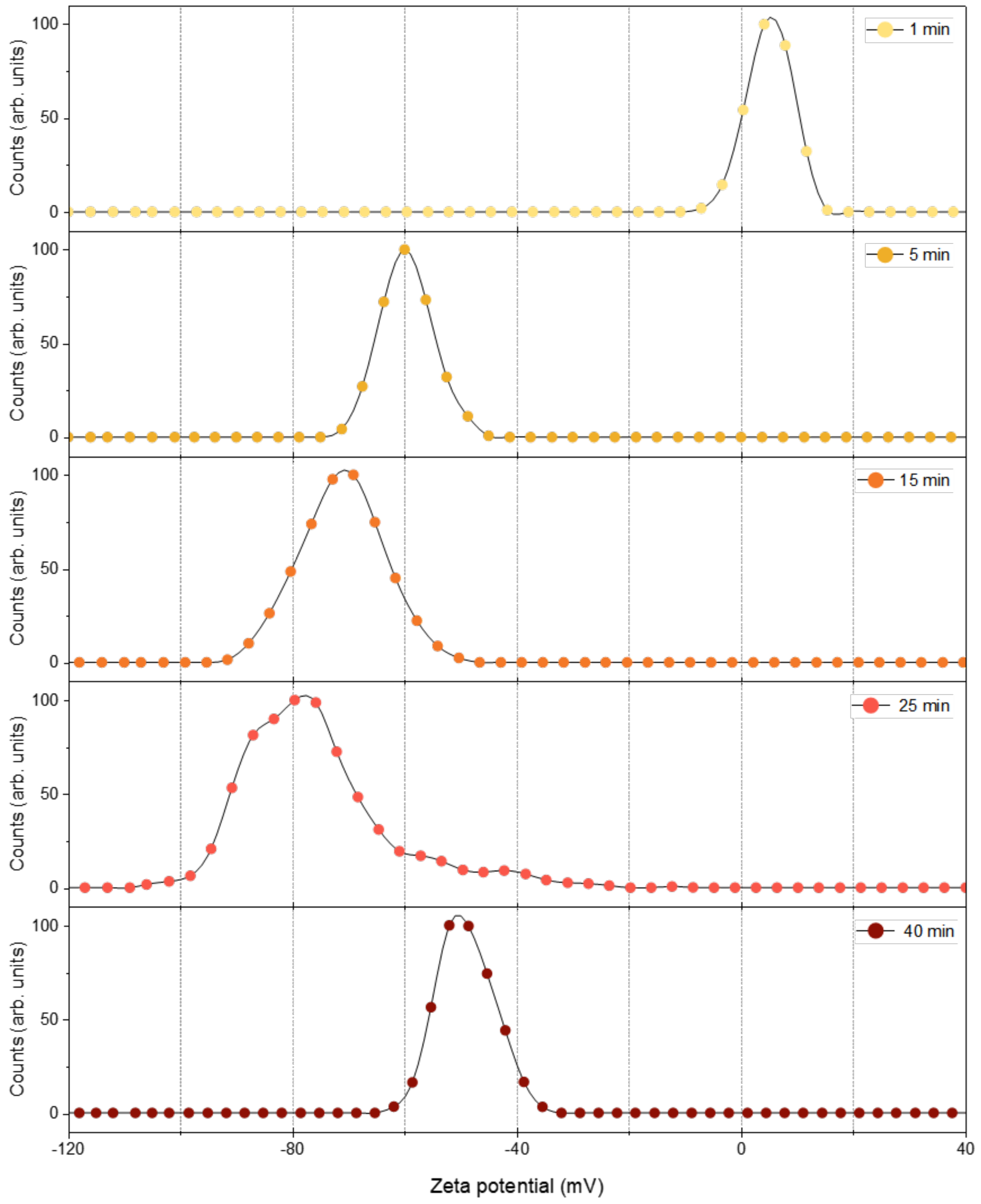

Supplementary Figure 2. Zeta potential distributions of emulsion with various sonication time.

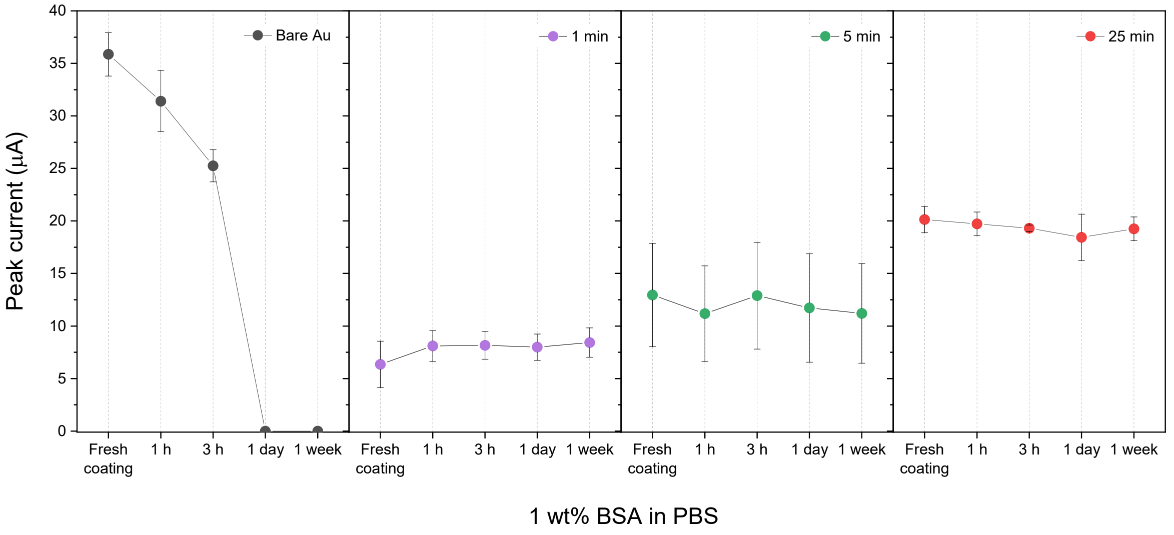

Supplementary Figure 3. Comparison of mean peak current values recorded at the bare gold electrodes and emulsion-based nanocomposites with various sonication time. Data are presented as mean values ± SD (n = 4 independent experiments). Sensors were incubated in 1% BSA solution. Emulsion-based nanocomposites sonicated for 25 min showed the highest uniformity in electrochemical signal for a week.

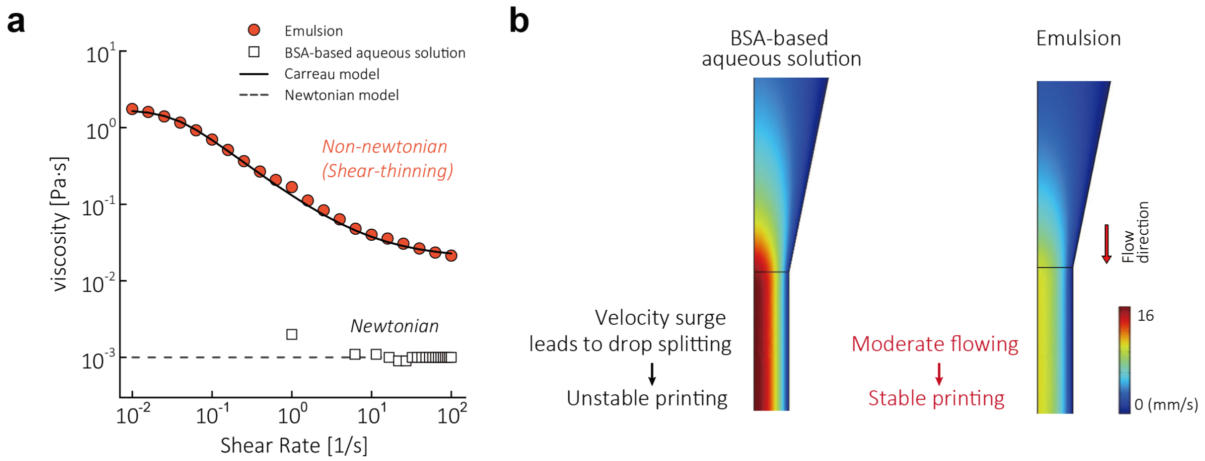

Supplementary Figure 4. a, Measurement of rheological properties for emulsion and BSA-based aqueous solution (control). Solid line represents Carreau model for emulsion and dashed line indicates Newtonian model for control. **b,** CFD simulation of flow behavior at the nozzle tip using emulsion and aqueous solution (control). For aqueous solution, inviscid flow causes velocity surge at the nozzle tip, leading to drop splitting and unstable printing performance. On the other hand, the emulsion-based printing shows moderate flowing at the tip due to its unique shear thinning feature, which results in high uniformity of patterning. Colormap shows the velocity field in mm s^{-1}. Outlet diameter is 0.25 mm.

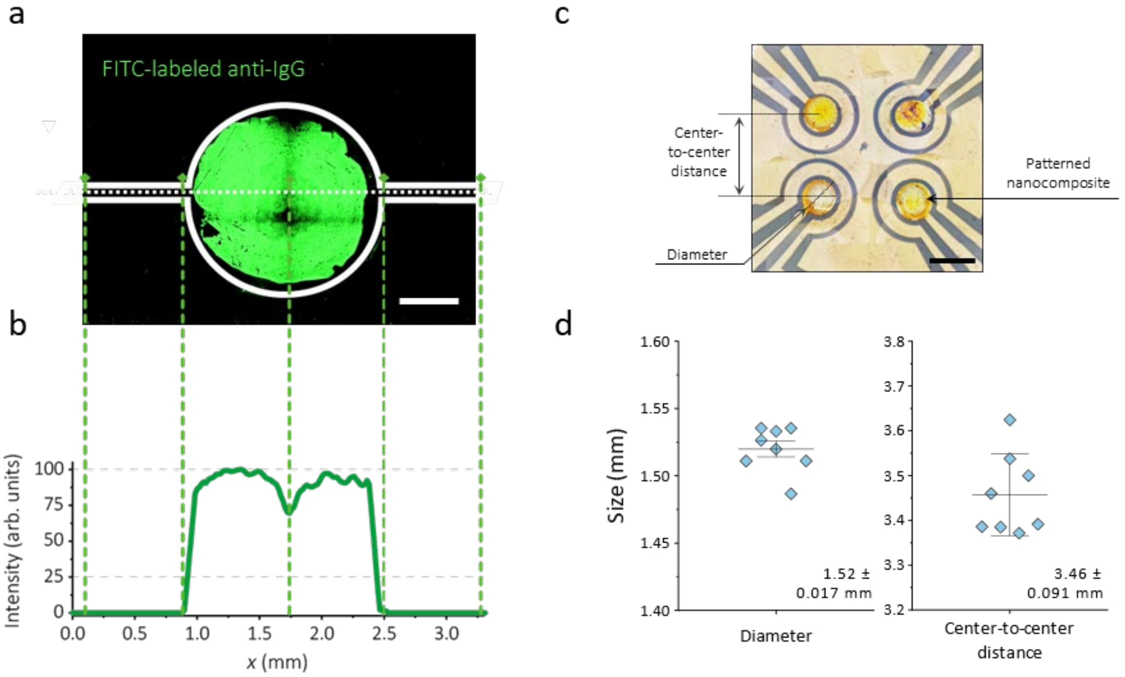

Supplementary Figure 5. a, Confocal microscopy image of patterned emulsion-based nanocomposite immobilized with FITC-labeled anti-IgG at excitation wavelength of 488 nm. Scale bar is 500 µm. **b,** Intensity of immobilized FITC-labeled anti-IgG along the *x*-axis (white dashed line). The diameter of pattern showing high intensity is around 1.5 mm which corresponds to the designed diameter of a single working electrode. Coefficient of variation (CV) for intensity within the printed composite was 7.81%. Data reproducibility was confirmed by two independent experiments. **c**, An optical microscopy image of the patterned emulsion-based nanocomposites on the working electrodes. Scale bar is 1.5 mm. **d,** Size distribution of diameter (left) and center-to-center distance (right) of the patterned nanocomposites. Data represents as mean values ± SD (n = 8 independent experiments). The CV for the diameter and center-to-center distance were 1.10% and 2.64%, respectively.

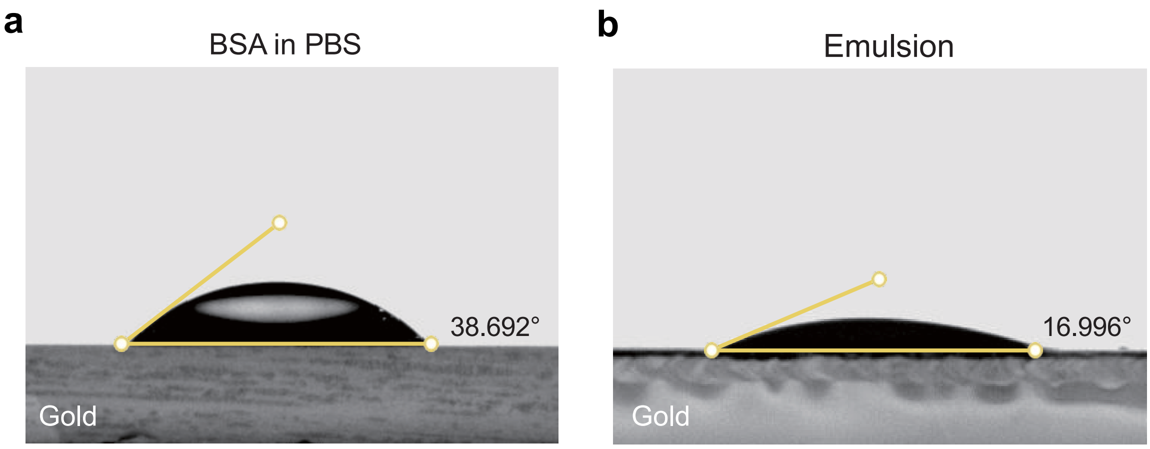

Supplementary Figure 6. a, Contact angles of PBS containing BSA and **b,** emulsion on the gold substrate. The smaller the contact angle value, the better the wettability of the solution and the better the adherence to the substrate. Data reproducibility was confirmed by two independent experiments.

a

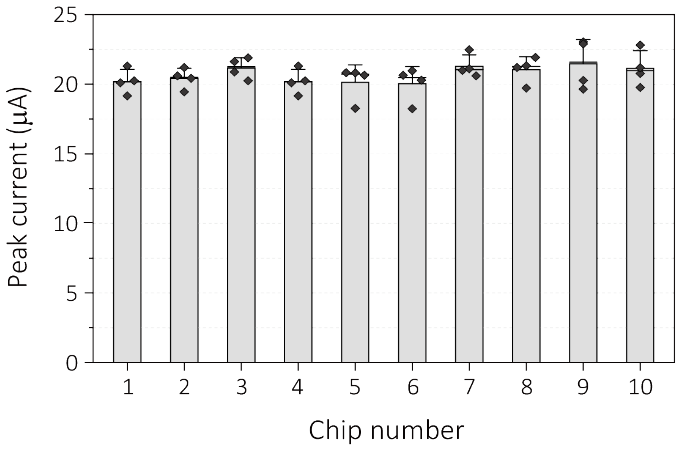

b

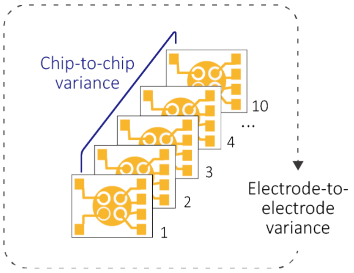

	emulsion-based sensor
$CV_{electrode\text{-}to\text{-}electrode}$	5.3%
$CV_{chip\text{-}to\text{-}chip}$	5.37%

Supplementary Figure 7. a, Analysis of chip-to-chip variation of emulsion-based sensor determined by anodic peak currents with a potassium ferro/ferricyanide solution. Data represents as mean values ± SD (n = 4 independent experiments). **b,** The coefficient of variations for electrode-to-electrode ($CV_{electrode\text{-}to\text{-}electrode}$) and chip-to-chip variations ($CV_{chip\text{-}to\text{-}chip}$) are 5.3% and 5.37%, respectively.

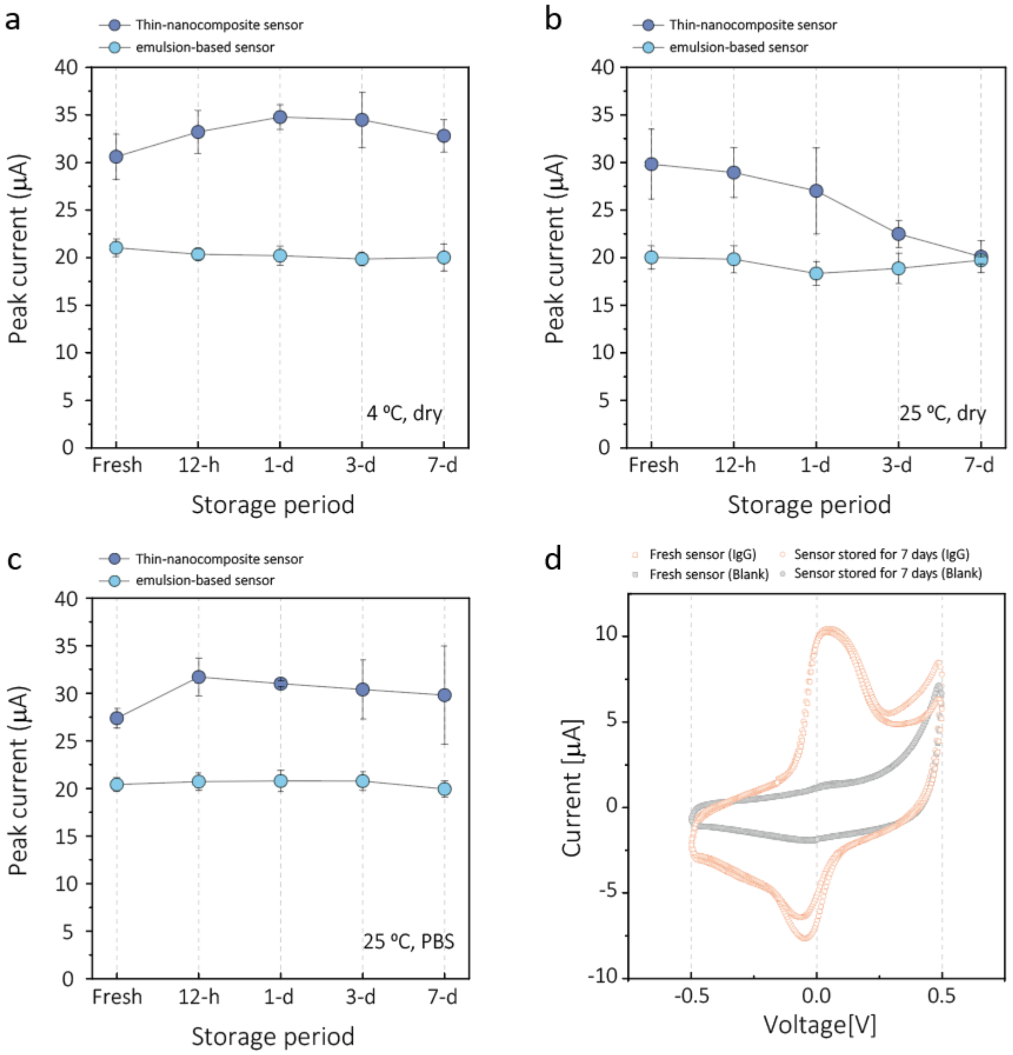

Supplementary Figure 8. a-c, Shelf-life of the emulsion-based sensor was evaluated under dry state and N_2 atmosphere at 4 °C and 25 °C, as well as at 25 °C in PBS. Thin nanocomposite sensors were tested as control. Over a storage period of 7 days, the emulsion-based sensor maintained a peak current at approximately 20 μA, with retention values of 95.34 % at 4 °C and 98.75 % at 25 °C. These values were similar to those observed when stored in PBS (4 °C: 98.75 % and 25 °C: 97.71 %). Thin nanocomposite sensor exhibited relatively consistent electrochemical performance (retention value: 107.70 %) at 4 °C but show vulnerability to 25 °C with a retention value of 68.01 %. Therefore, it was determined that storage under dry conditions at 25 °C provided the best performance for emulsion-based sensors. Data represents as mean values ± SD (n = 4 independent experiments). **d,** Sensor performance of emulsion-based sensor in detecting 100 pg mL^{-1} IgG after being stored in a dry state at 25 °C for 7 days.

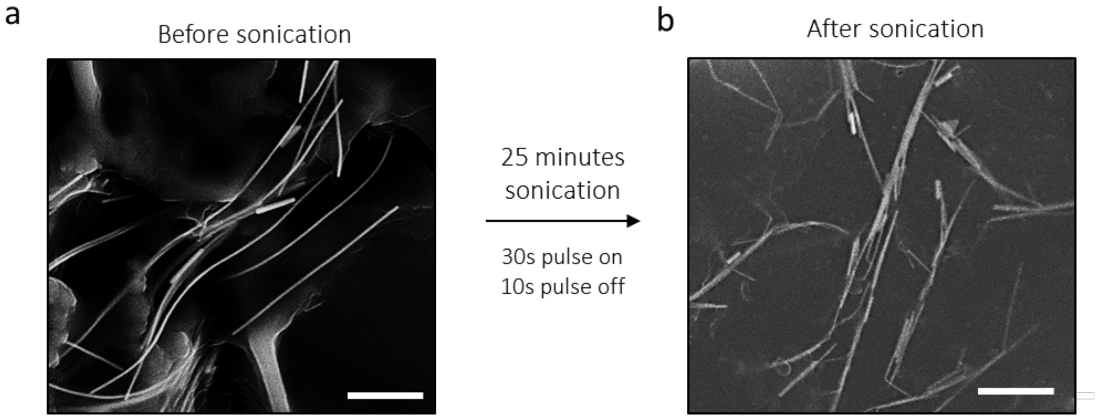

Supplementary Figure 9. SEM images of the gold nanowires **a,** before and **b,** after sonicating for 25 minutes. There was no significant snapping or breakage of the gold nanowire after sonication. Scale bars are 2 μm. Data reproducibility was confirmed by two additional experiments.

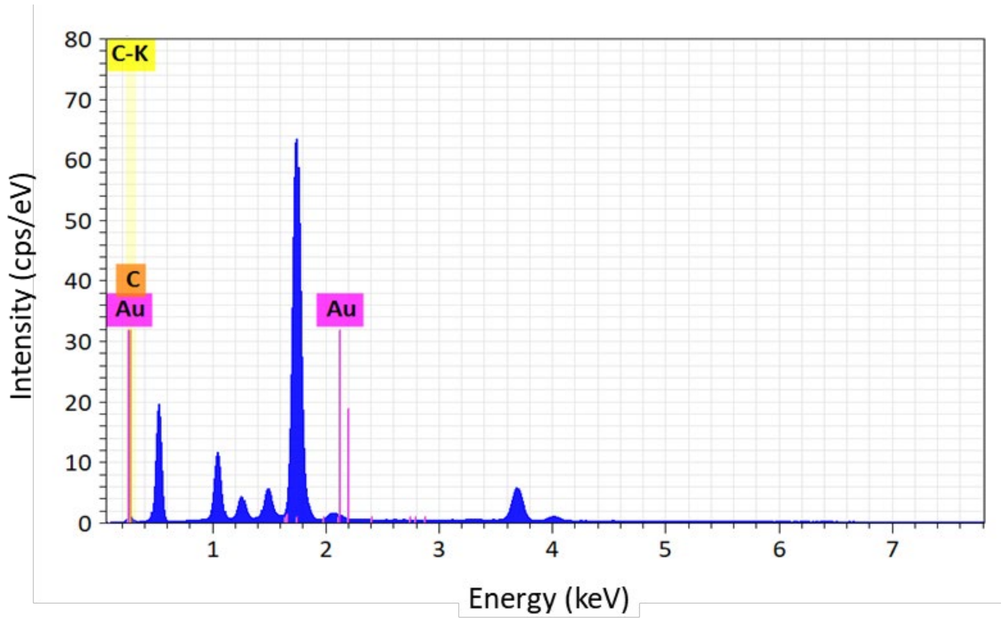

Supplementary Figure 10. Energy-dispersive X-ray spectroscopy (EDS) analysis of the emulsion-based nanocomposite. Au peak indicates the AuNWs embedded in the cross-linked BSA matrix.

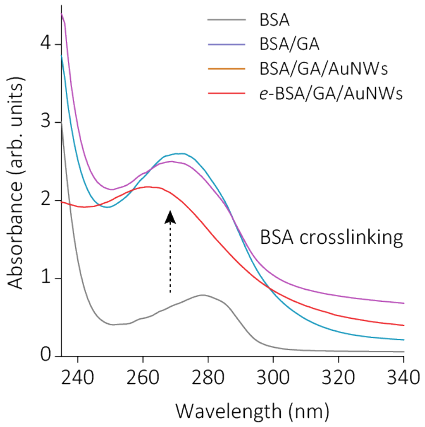

Supplementary Figure 11. UV-vis absorbance spectra of solutions with various components. BSA proteins were cross-linked by glutaraldehyde (GA), as indicated by the increase in absorbance at 265-270 nm.

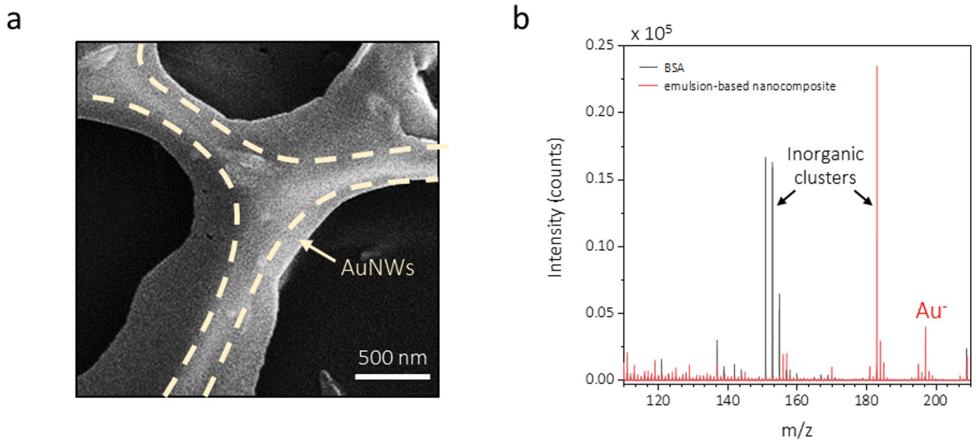

Supplementary Figure 12. Structural integrity of AuNWs within the porous nanocomposite. **a**, SEM images of emulsion-processed nanocomposite. AuNWs were observed to form a porous nanocomposite, encapsulated by BSA. Data reproducibility was confirmed by two independent experiments. **b**, Negative TOF-SIMS spectra of Au electrode with BSA and emulsion-based nanocomposite. *y*-axis denotes the detected number of secondary ions and *x*-axis denotes the mass-to-charge (m/z) ratio. Au^- signal in the spectrum of emulsion-based nanocomposite was originated from AuNWs. This result clearly revealed Au peak in the nanocomposite that were not observed in BSA control, a result corroborated by EDS analysis in the Fig. 2e and Supplementary Fig. 10.

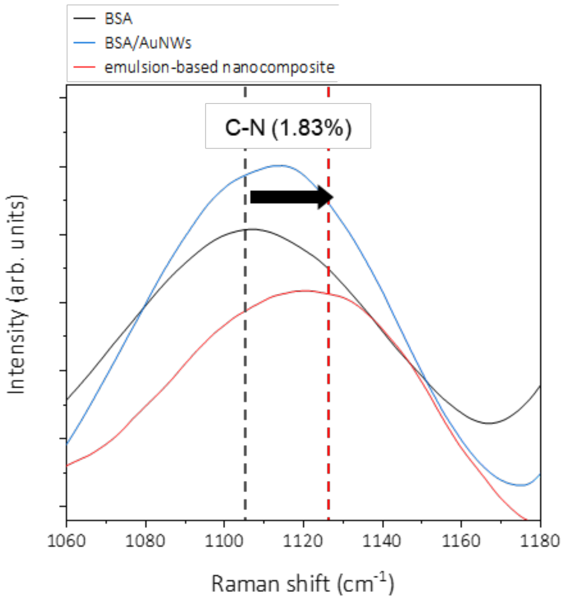

Supplementary Figure 13. Raman spectra of BSA, BSA/AuNWs, and emulsion-based nanocomposite. C-N peaks of BSA and BSA/AuNWs were 1110.35 cm^{-1} and that of emulsion-based nanocomposite was 1120.43 cm^{-1}. 1.83% shift of C-N peak indicates the peptide bonding formation in emulsion-based nanocomposite.

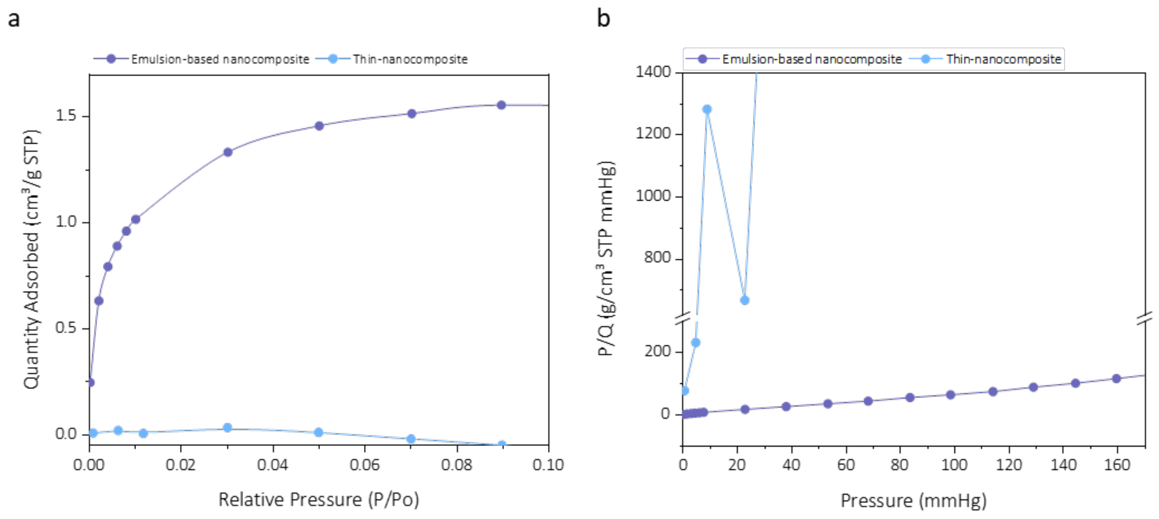

Supplementary Figure 14. N_2 physisorption analysis of thin and emulsion-based nanocomposite. **a,** BET surface area is calculated from the slope and the intercept according to the equation of the linear range. BET surface area of emulsion-based nanocomposite is about 38.7-fold higher than that of thin nanocomposite. ($S_{BET, thin\ nanocomposite}$: 0.1677 m^2/g, $S_{BET, emulsion-based\ nanocomposite}$: 6.4859 m^2/g.) **b,** Langmuir surface area of emulsion-based nanocomposite is about 38.7-fold higher than that of thin nanocomposite. Langmuir surface area of the thin nanocomposite is not fitted. ($S_{Langmuir, thin\ nanocomposite}$: 0.177 m^2/g, $S_{Langmuir, emulsion-based\ nanocomposite}$: 6.8526 m^2/g.)

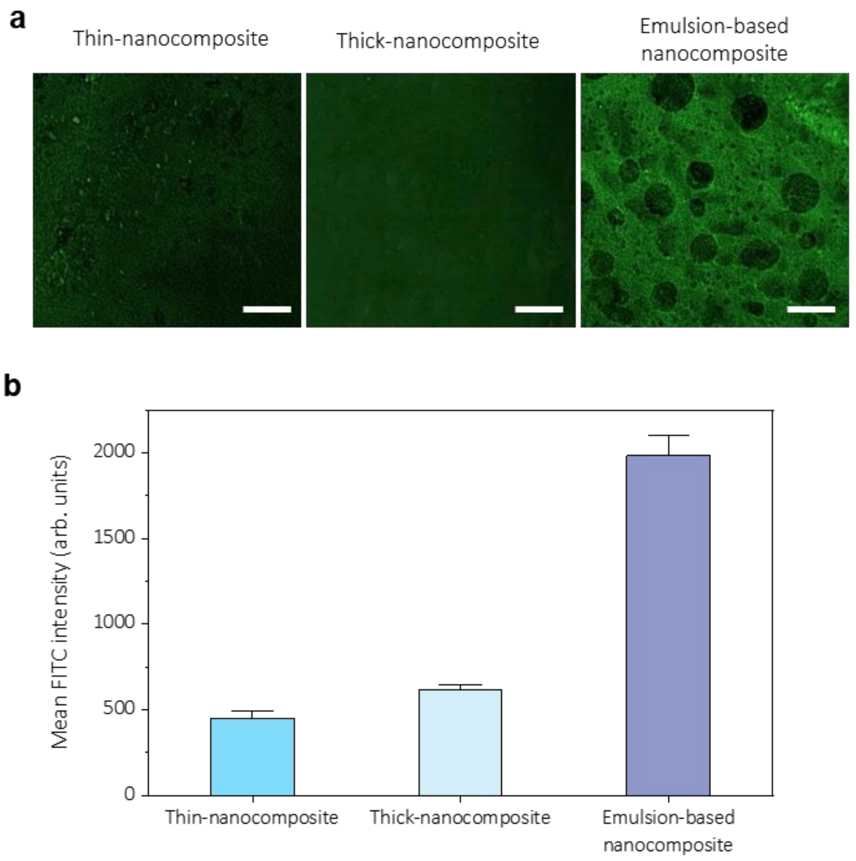

Supplementary Figure 15. Nanocomposites immobilized with FITC-labeled anti-IgG. **a,** Confocal microscopy images of thin, thick, and emulsion-based nanocomposite. All nanocomposites were imaged with an excitation wavelength 488 nm for FITC. Scale bars represent 1 µm. Data reproducibility was confirmed by two independent experiments. **b,** Fluorescence intensity variation of thin, thick, and emulsion-based nanocomposites. Image of the emulsion-based nanocomposites showed 4.41 and 3.21-fold higher than those of thin and thick nanocomposite due to its increased surface area. Data represents as mean values ± SD (n = 4 independent experiments).

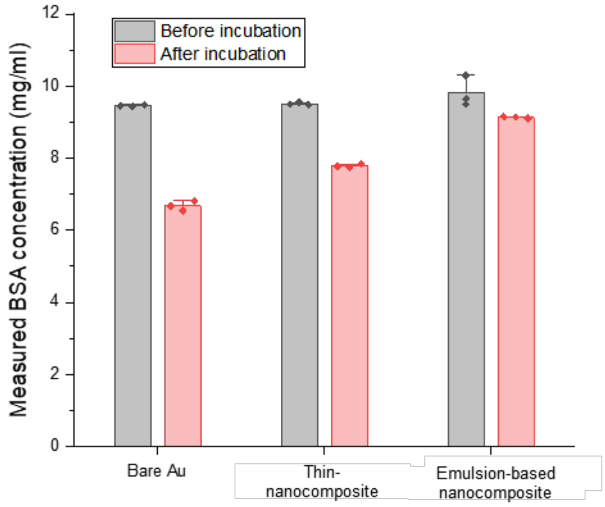

Supplementary Figure 16. BSA concentration change before (gray) and after (red) the incubation of sensors in a 1% BSA solution for one day. Data represents as mean values ± SD (n = 3 independent experiments). Concentration was calculated using Nanodrop at a wavelength of 280 nm. For bare Au, 70.5% of BSA was measured after incubation. For thin nanocomposite sensor, 81.8% of BSA was measured after incubation. For emulsion-based nanocomposite, 93% of BSA was measured after incubation, demonstrating superior antifouling properties.

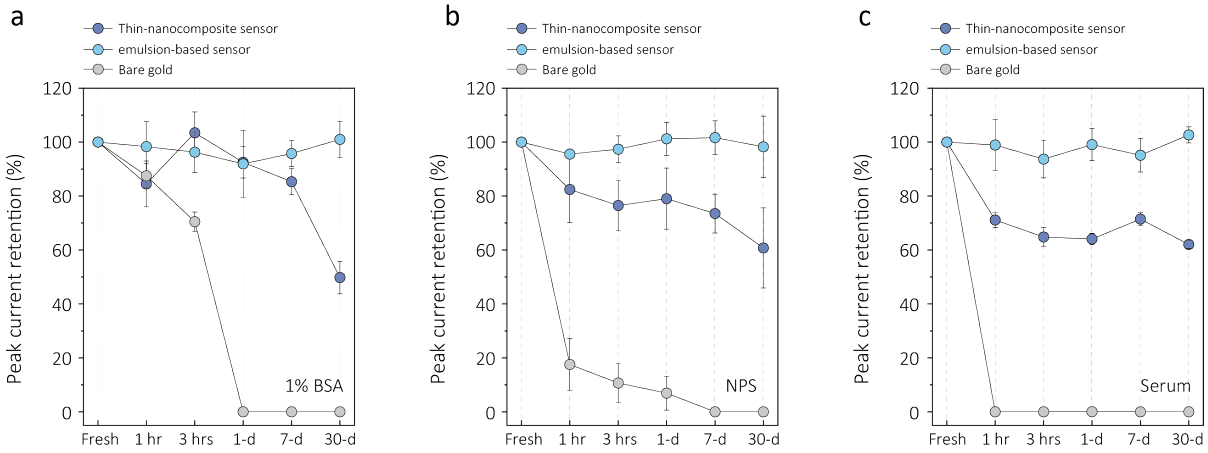

Supplementary Figure 17. Peak current retention of the emulsion-based sensor, the thin nanocomposite sensor, and bare gold electrodes under incubating with **a,** 1% BSA, **b,** NPS, and **c,** serum. The emulsion-based sensor showed retention of 101.01% (1% BSA), 98.25% (NPS), and 102.67% (serum). Data represents as mean values ± SD (n = 4 independent experiments).

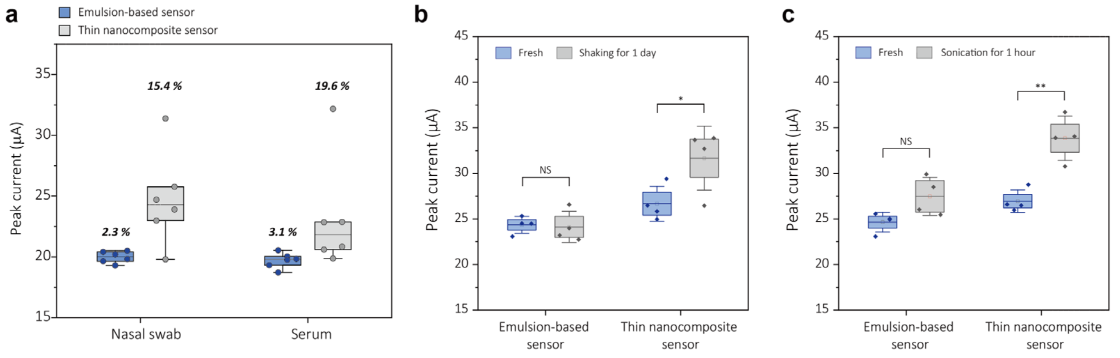

Supplementary Figure 18. a, Signal drift analysis for emulsion-based sensors and thin nanocomposite sensors when exposed to human nasal swab and serum over one month. The emulsion sensor exhibited a CV of 2.3% for nasal swab and 3.1% for serum, while the thin nanocomposite sensor showed higher signal drift with CV of 15.4% and 19.6%, respectively. Each data represents the average values from four electrodes and was measured at intervals of fresh, 1 hour, 3 hours, 1 day, 1 week, and 1 month. **b** and **c**, Durability test under the mechanical forces. Both sensors were immersed in PBS and exposed to an orbital shaker at 150 rpm (**b**) and the bath sonication for one hour (**c**), respectively. In the boxes, the 25th, median values, and 75th percentiles of the data are indicated. The whiskers denote 1.5× interquartile range (IQR). Statistical significance was tested (*P < 0.05, **P < 0.01; two-tailed Student's t-test).

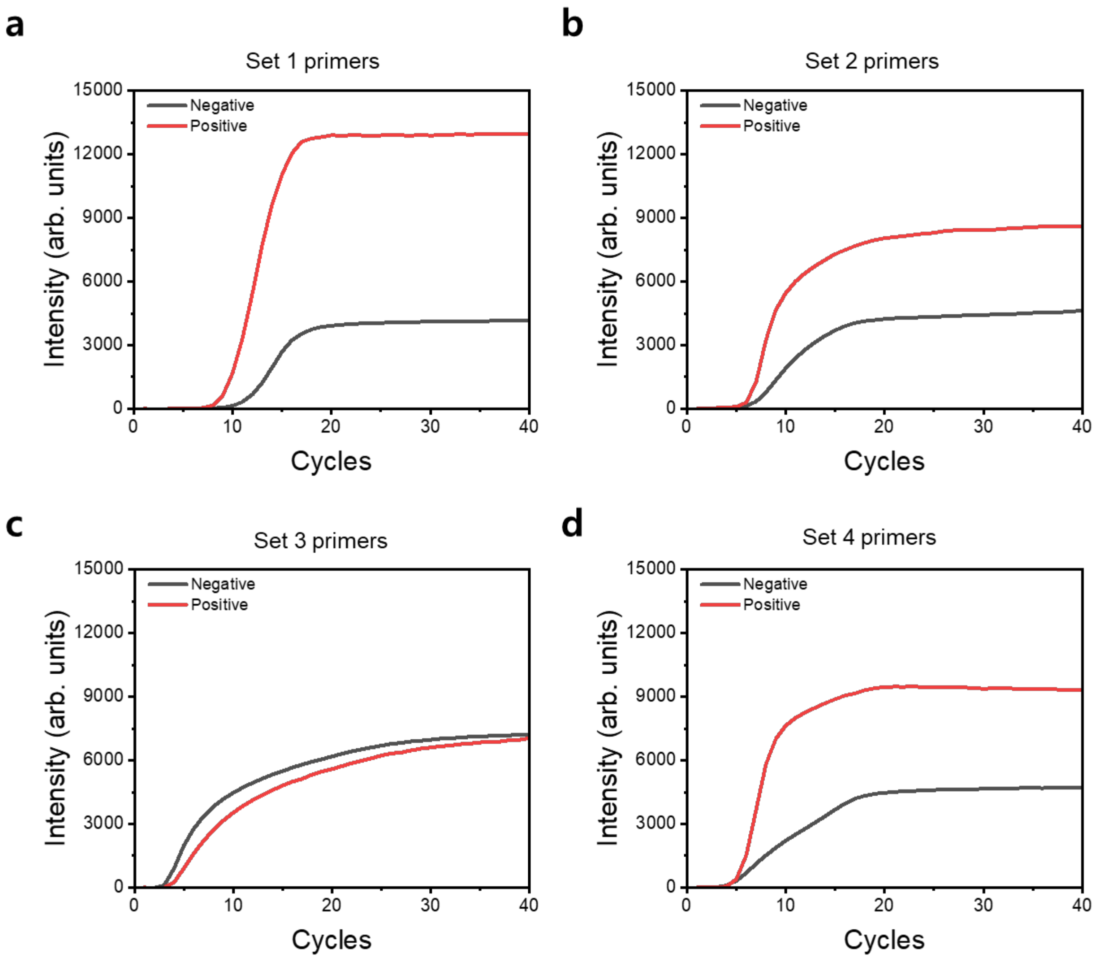

Supplementary Figure 19. a-d, Evaluation of RT-RPA primers for ORF1a gene of SARS-CoV-2. Set 1 primers showed the highest signal ratio between positive and negative samples.

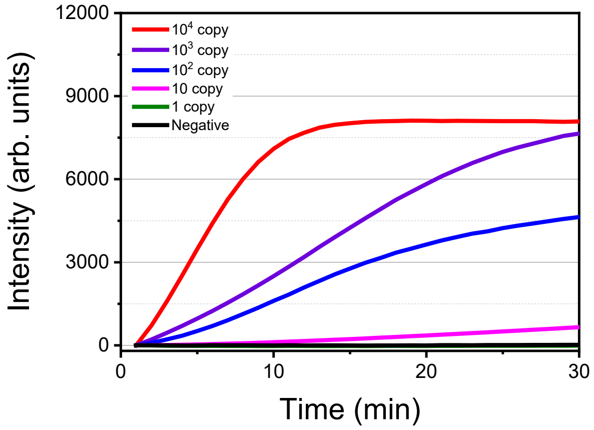

Supplementary Figure 20. Sensitivity of the RT-RPA assay. Time-dependent fluorescence intensities during RT-RPA assay with various concentrations of ORF1a gene in SARS-CoV-2 RNA. [ORF1a primers] = 500 nM, [M-MLV reverse transcriptase] = 20 U, [Murine RNase inhibitor]= 60 U, and [MgOAc] = 15 mM.

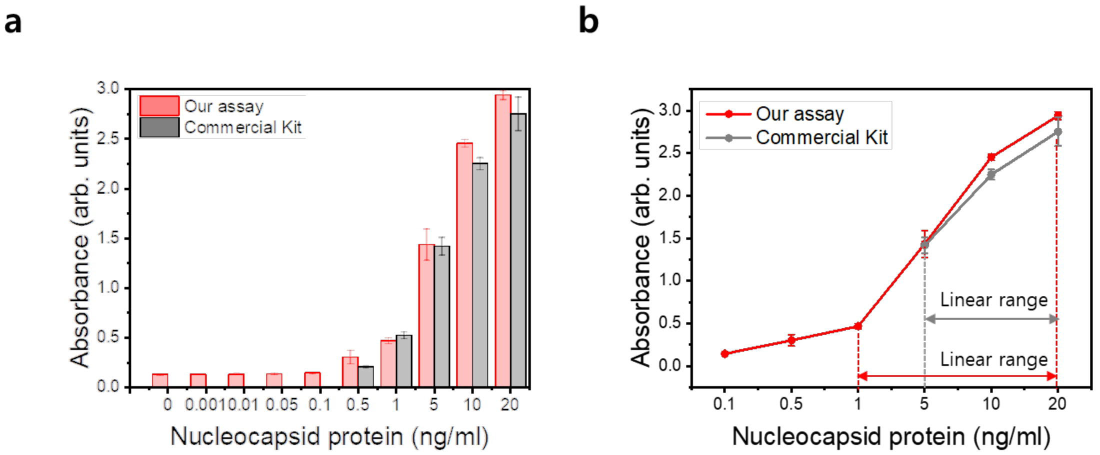

Supplementary Figure 21. Comparison of the results for our assay with conventional ELISA kit. **a,** Absorbance intensities at 450 nm in the presence of various concentrations of SARS-CoV-2 nucleocapsid protein with our assay and conventional ELISA kit. **b,** The linear range of our assay compared to conventional ELISA kit for target nucleocapsid protein. Data represents as mean values ± SD (n = 3 independent experiments). [Capture antibody] = 0.1 µg μl^{-1} and [Detection antibody] = 0.25 µg μl^{-1}.

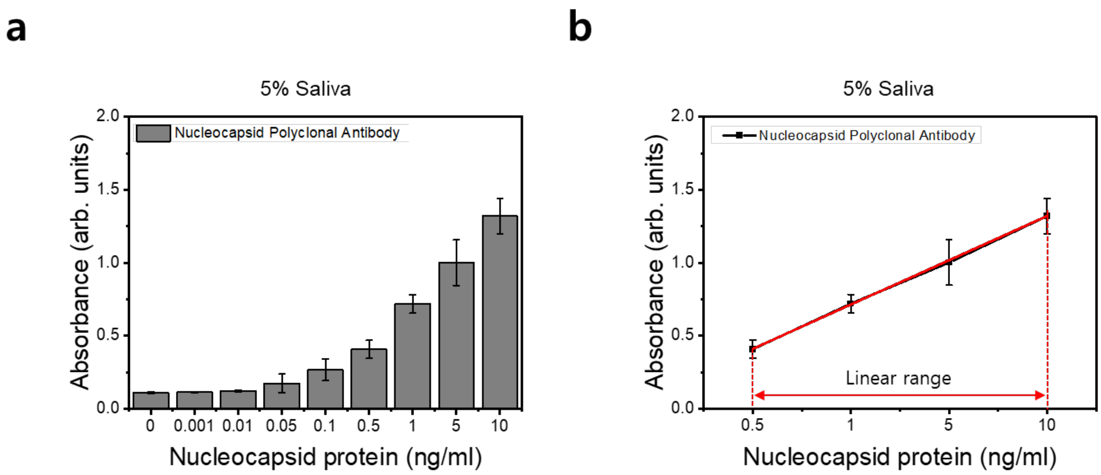

Supplementary Figure 22. Determination of SARS-CoV-2 nucleocapsid protein in diluted artificial saliva samples (5%). **a,** Absorbance intensities at 450 nm in the presence of various concentrations of SARS-CoV-2 nucleocapsid protein spiked in artificial saliva samples (5%). **b,** The linear range of our assay for target nucleocapsid protein spiked in artificial saliva samples (5%). Data represents as mean values ± SD (n = 3 independent experiments). [Capture antibody] = 0.1 μg μl^{-1} and [Detection antibody] = 0.25 μg μl^{-1}.

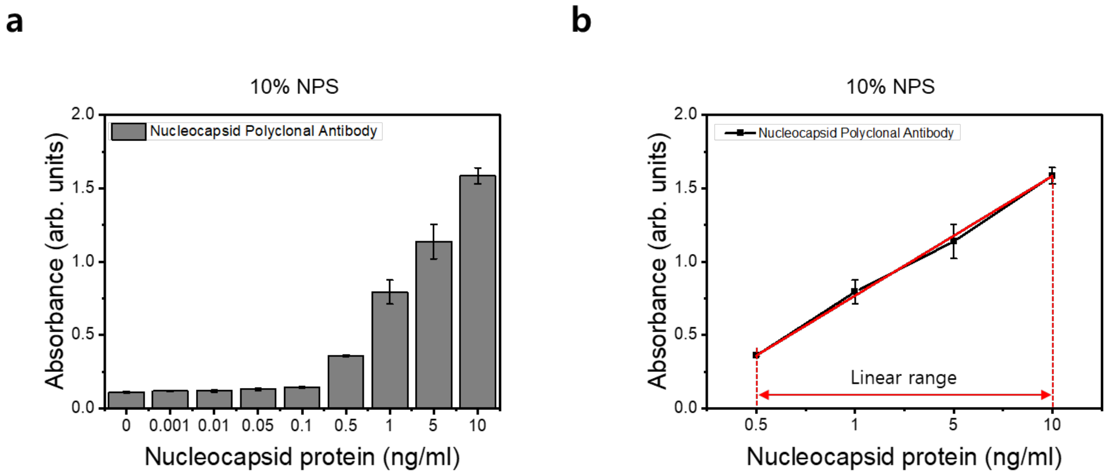

Supplementary Figure 23. Determination of SARS-CoV-2 nucleocapsid protein in diluted nasopharyngeal swabs (NPS) samples (10 %). **a,** Absorbance intensities at 450 nm in the presence of various concentrations of SARS-CoV-2 nucleocapsid protein spiked in NPS samples (10%). **b,** The linear range of our assay for target nucleocapsid protein spiked in NPS samples (10%). Data represents as mean values ± SD (n = 3 independent experiments). [Capture antibody] = 0.1 μg $μl^{-1}$ and [Detection antibody] = 0.25 μg $μl^{-1}$.

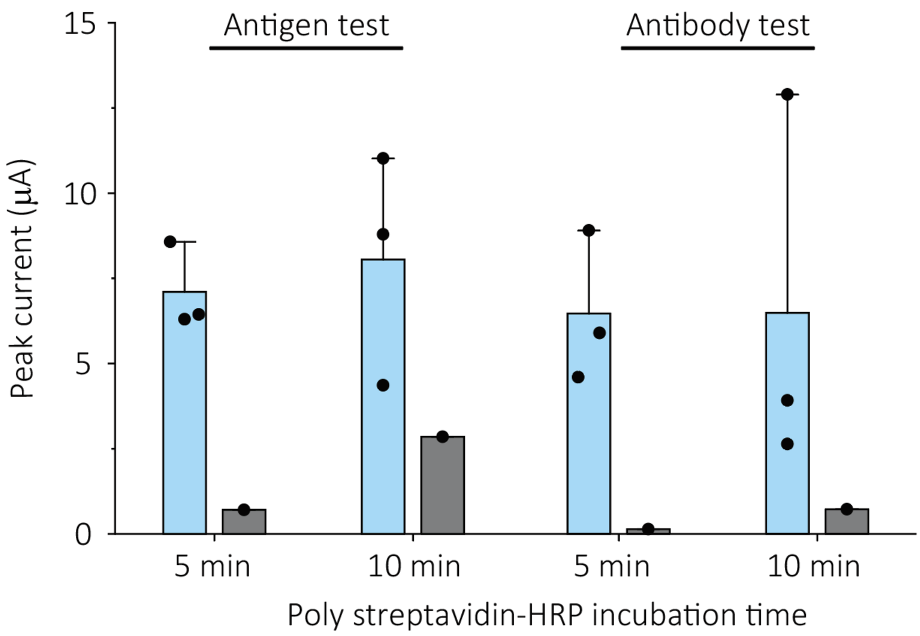

Supplementary Figure 24. Optimization of HRP incubation time for antigen and antibody detection using emulsion-based sensor. Data represents as mean values ± SD (n = 3 independent experiments) for positive samples (blue).

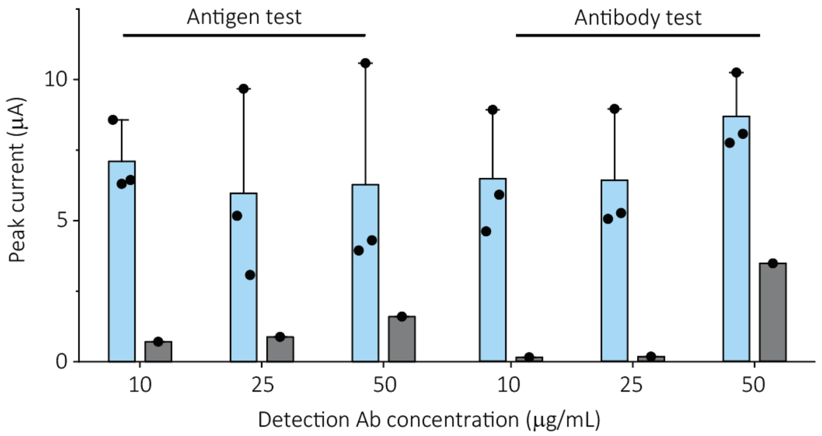

Supplementary Figure 25. Optimization of detection antibody concentration for antigen and antibody detection using emulsion-based sensor. Data represents as mean values ± SD (n = 3 independent experiments) for positive samples (blue).

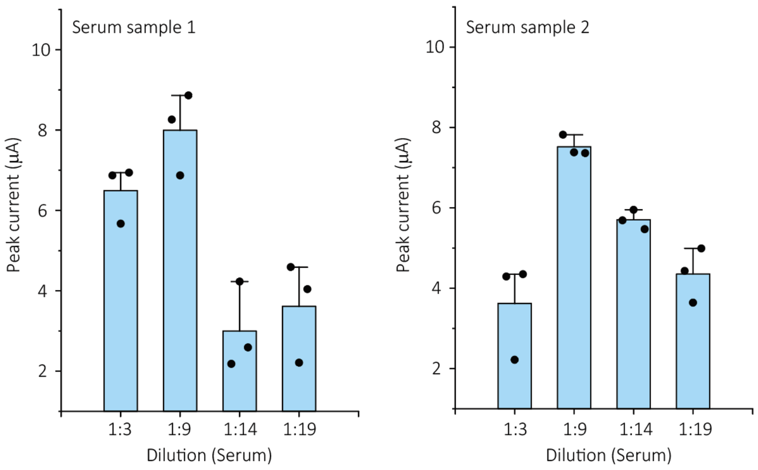

Supplementary Figure 26. Optimization of serum dilution ratio for antibody detection using emulsion-based sensor. Two COVID-19 positive serum samples were tested. Data represents as mean values ± SD (n = 3 independent experiments).

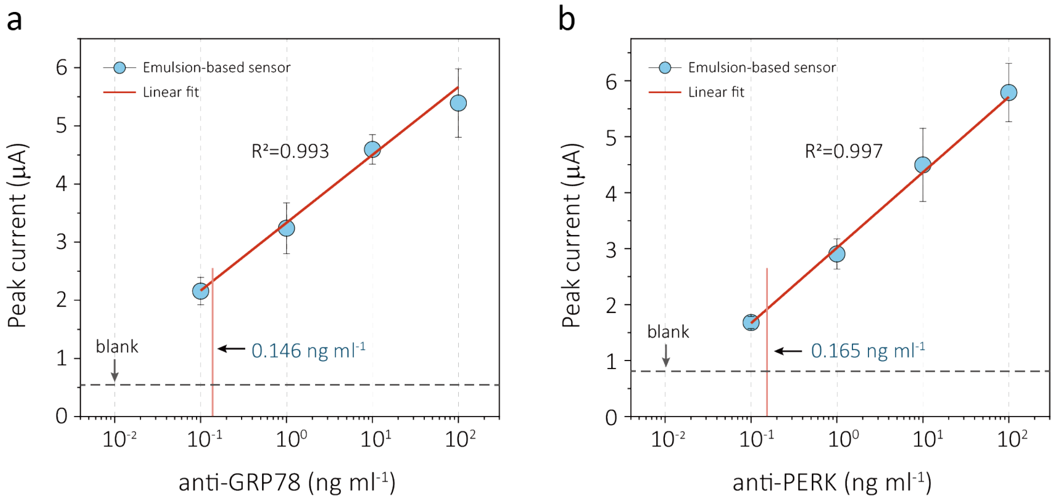

Supplementary Figure 27. a and b, Electrochemical detection of ER stress-related biomarkers, such as anti-GRP78 (**a**) and anti-PERK (**b**), using the emulsion-based sensor**.** Calibration curves were obtained for both anti-GRP78 and anti-PERK, covering a dynamic range from 100 pg mL^{-1} to 100 ng mL^{-1}, and exhibiting LOD of 0.146 ng mL^{-1} and 0.165 ng mL^{-1}, respectively. Anti-GRP78 and anti-PERK were spiked in PBS. LOD was defined using three standard deviations (3σ) of the blank solution. Signals from blank solution were illustrated as gray dashed line. Data represents as mean values ± SD (n = 4 independent experiments).

Supplementary Table 1. Sequences of oligonucleotide used in this experiment.

Target	Name	Sequence (5' → 3')[a]
ORF1a gene	Set 1 FP	AAA TTG TTA AAT TTA TCT CAA CCT GTG CTT GT
	Set 1 RP	AGT TTC TTC TCT GGA TTT AAC ACA CTT TCT
	Set 2 FP	GCT TGT GAA ATT GTC GGT GGA CAA ATT GTC ACC TG
	Set 2 RP	CTC CAC CAA TAA TGA TAG AGT CAG CAC ACA AAG CC
	Set 3 FP	TGA AAT TGT CGG TGG ACA AAT TGT CAC CTG TGC AA
	Set 3 RP	TCA CCT AAA TTC AAG GCT TTA AGT TTA GCT CCA CC
	Set 4 FP	TTG TCG GTG GAC AAA TTG TCA CCT GTG CAA AGG AA
	Set 4 RP	CCC TTT GAG TGC GTG ACA AAT GTT TCA CCT AAA TT
	guideRNA	AltR1/UAA UUU CUA CUC UUG UAG AUA GCU UGU AAA UAA AUU UUU GGC UU /AltR2/
	PNA capture probe	Amine - ACA ACA ACA ACA ACA
	Reporter probe	Biotin – ATT ATT ATT ATT ATT TGT TGT TGT TGT TGT

Supplementary Table 2. Diagnostic results of 60 clinical samples (positive: 40, negative:20) by qRT-PCR and our electrochemical assay for detection of SARS-CoV-2 ORF1a gene. Cut-off current value was determined from ROC curves: 2.12 μA.

Number	Sample type	C_t value			Peak current	
		RdRP	E	P/N	Result	P/N
1	NPS	18.26	19.25	P	0.66	P
2	NPS	21.06	21.23	P	0.91	P
3	NPS	13.72	15.13	P	0.34	P
4	NPS	13.86	14.42	P	0.09	P
5	NPS	19.65	16.41	P	0.18	P
6	NPS	25.24	25.27	P	0.74	P
7	NPS	19.21	19.56	P	0.01	P
8	NPS	23.79	20.82	P	0.83	P
9	NPS	22.02	20.11	P	1.06	P
10	NPS	18.37	16.37	P	0.34	P
11	NPS	16.83	18.02	P	0.19	P
12	NPS	28.82	30.81	P	0.01	P
13	NPS	20.04	21.4	P	0.06	P
14	NPS	21.29	20.14	P	0.20	P
15	NPS	13.04	13.33	P	0.20	P
16	NPS	25.37	25.96	P	0.17	P
17	NPS	14.5	15.31	P	0.44	P
18	NPS	18.62	19.2	P	0.70	P
19	NPS	18.45	19.24	P	0.06	P
20	NPS	15.42	16.38	P	0.05	P
21	NPS	13.1	13.91	P	0.05	P

Number	Sample type	C_t value			Peak current	
		RdRP	E	P/N	Result	P/N
22	NPS	19.38	21.04	P	0.00	P
23	NPS	16.67	18.47	P	0.00	P
24	NPS	9.38	11.52	P	0.00	P
25	NPS	15.29	16.94	P	0.00	P
26	NPS	13.3	14.56	P	0.00	P
27	NPS	19.15	19	P	0.50	P
28	NPS	14.25	15.1	P	0.31	P
29	NPS	13.73	14.02	P	0.04	P
30	NPS	16.8	16.39	P	0.00	P
31	NPS	11.59	11.89	P	0.00	P
32	NPS	25.2	26.27	P	0.00	P
33	NPS	14.09	13.58	P	0.05	P
34	NPS	18.56	18.8	P	0.00	P
35	NPS	17.74	17.48	P	0.00	P
36	NPS	22.14	21.08	P	0.03	P
37	NPS	30.75	29.74	P	0.22	P
38	NPS	31.73	32.31	P	0.00	P
39	NPS	17.02	16.25	P	0.00	P
40	NPS	22.67	23.32	P	0.00	P
41	NPS	N/A	N/A	N	4.39	N
42	NPS	N/A	N/A	N	5.42	N
43	NPS	N/A	N/A	N	6.65	N
44	NPS	N/A	N/A	N	3.20	N
45	NPS	N/A	N/A	N	4.85	N

Number	Sample type	C_t value			Peak current	
		RdRP	E	P/N	Result	P/N
46	NPS	N/A	N/A	N	7.31	N
47	NPS	N/A	N/A	N	7.75	N
48	NPS	N/A	N/A	N	7.07	N
49	NPS	N/A	N/A	N	6.92	N
50	NPS	N/A	N/A	N	6.33	N
51	NPS	N/A	N/A	N	10.17	N
52	NPS	N/A	N/A	N	6.70	N
53	NPS	N/A	N/A	N	7.93	N
54	NPS	N/A	N/A	N	6.24	N
55	NPS	N/A	N/A	N	10.97	N
56	NPS	N/A	N/A	N	7.68	N
57	NPS	N/A	N/A	N	8.55	N
58	NPS	N/A	N/A	N	9.98	N
59	NPS	N/A	N/A	N	10.84	N
60	NPS	N/A	N/A	N	10.87	N

Supplementary Table 3. Diagnostic results of 60 clinical samples (positive: 40, negative:20) by qRT-PCR and our electrochemical assay for detection of SARS-CoV-2 nucleocapsid protein. Cut-off current value was determined from ROC curves: 0.857 µA.

Number	Sample type	C_t value			Peak current	
		RdRP	E	P/N	Result	P/N
1	NPS	18.26	19.25	P	12.30	P
2	NPS	21.06	21.23	P	7.08	P
3	NPS	13.72	15.13	P	8.19	P
4	NPS	13.86	14.42	P	12.79	P
5	NPS	19.65	16.41	P	9.58	P
6	NPS	25.24	25.27	P	3.56	P
7	NPS	19.21	19.56	P	5.16	P
8	NPS	23.79	20.82	P	3.62	P
9	NPS	22.02	20.11	P	6.69	P
10	NPS	18.37	16.37	P	7.93	P
11	NPS	16.83	18.02	P	8.00	P
12	NPS	28.82	30.81	P	3.70	P
13	NPS	20.04	21.4	P	6.03	P
14	NPS	21.29	20.14	P	3.14	P
15	NPS	13.04	13.33	P	9.65	P
16	NPS	25.37	25.96	P	2.83	P
17	NPS	14.5	15.31	P	7.34	P
18	NPS	18.62	19.2	P	4.62	P
19	NPS	18.45	19.24	P	2.94	P
20	NPS	15.42	16.38	P	4.59	P
21	NPS	13.1	13.91	P	8.56	P
22	NPS	19.38	21.04	P	6.50	P

Number	Sample type	C_t value			Peak current	
		RdRP	E	P/N	Result	P/N
23	NPS	16.67	18.47	P	13.27	P
24	NPS	9.38	11.52	P	10.83	P
25	NPS	15.29	16.94	P	3.62	P
26	NPS	13.3	14.56	P	10.06	P
27	NPS	19.15	19	P	8.84	P
28	NPS	14.25	15.1	P	11.39	P
29	NPS	13.73	14.02	P	9.12	P
30	NPS	16.8	16.39	P	6.31	P
31	NPS	11.59	11.89	P	6.58	P
32	NPS	25.2	26.27	P	2.96	P
33	NPS	14.09	13.58	P	7.36	P
34	NPS	18.56	18.8	P	12.19	P
35	NPS	17.74	17.48	P	7.25	P
36	NPS	22.14	21.08	P	3.56	P
37	NPS	30.75	29.74	P	0.80	N
38	NPS	31.73	32.31	P	0.89	P
39	NPS	17.02	16.25	P	3.60	P
40	NPS	22.67	23.32	P	6.34	P
41	NPS	N/A	N/A	N	0.61	N
42	NPS	N/A	N/A	N	0.69	N
43	NPS	N/A	N/A	N	0.02	N
44	NPS	N/A	N/A	N	0.28	N
45	NPS	N/A	N/A	N	0.29	N
46	NPS	N/A	N/A	N	0.21	N

Number	Sample type	C_t value			Peak current	
		RdRP	E	P/N	Result	P/N
47	NPS	N/A	N/A	N	0.00	N
48	NPS	N/A	N/A	N	0.00	N
49	NPS	N/A	N/A	N	0.00	N
50	NPS	N/A	N/A	N	0.00	N
51	NPS	N/A	N/A	N	0.00	N
52	NPS	N/A	N/A	N	0.00	N
53	NPS	N/A	N/A	N	0.00	N
54	NPS	N/A	N/A	N	0.13	N
55	NPS	N/A	N/A	N	0.00	N
56	NPS	N/A	N/A	N	0.18	N
57	NPS	N/A	N/A	N	0.19	N
58	NPS	N/A	N/A	N	0.82	N
59	NPS	N/A	N/A	N	0.95	P
60	NPS	N/A	N/A	N	0.00	N

Supplementary Table 4. Diagnostic results of 53 clinical samples (positive: 33, negative: 20) by qRT-PCR and our electrochemical assay for detection of IgG. NPS were used to extract the Ct values. Cut-off current value was determined from ROC curves: 1.3 μA.

Number	Sample type	C_t value			Sample type	Peak current	
		RdRP	E	P/N		Result	P/N
1	NPS	15.7	17.17	P	Serum	5.53	P
2	NPS	23.81	25.37	P	Serum	5.20	P
3	NPS	19.41	19.25	P	Serum	4.08	P
4	NPS	19.68	19.49	P	Serum	1.51	P
5	NPS	19.96	20.71	P	Serum	5.47	P
6	NPS	18.73	19.26	P	Serum	5.50	P
7	NPS	24.15	24.26	P	Serum	6.80	P
8	NPS	12.48	13.02	P	Serum	4.76	P
9	NPS	24.55	24.37	P	Serum	2.79	P
10	NPS	24.89	23.99	P	Serum	6.61	P
11	NPS	23.11	23.45	P	Serum	4.32	P
12	NPS	23.65	21.16	P	Serum	2.60	P
13	NPS	15.01	14.45	P	Serum	6.41	P
14	NPS	17.49	17.99	P	Serum	5.16	P
15	NPS	35.97	34.92	P	Serum	7.17	P
16	NPS	22.84	21.46	P	Serum	5.01	P
17	NPS	8.55	-	P	Serum	4.26	P
18	NPS	22.91	23.56	P	Serum	4.98	P
19	NPS	27.91	27.01	P	Serum	8.54	P
20	NPS	15.7	17.17	P	Serum	0.85	N
21	NPS	15.7	17.17	P	Serum	5.62	P

Number	Sample type	C_t value			Sample type	Peak current	
		RdRP	E	P/N		Result	P/N
22	NPS	23.81	25.37	P	Serum	6.49	P
23	NPS	23.81	25.37	P	Serum	3.61	P
24	NPS	23.81	25.37	P	Serum	5.62	P
25	NPS	23.81	25.37	P	Serum	6.07	P
26	NPS	23.81	25.37	P	Serum	3.42	P
27	NPS	18.73	19.26	P	Serum	5.46	P
28	NPS	28.76	27.24	P	Serum	5.37	P
29	NPS	28.76	27.24	P	Serum	7.27	P
30	NPS	23.95	23.96	P	Serum	4.94	P
31	NPS	24.68	22.85	P	Serum	6.78	P
32	NPS	18.31	17.88	P	Serum	3.64	P
33	NPS	31.12	29.39	P	Serum	7.64	P
34	NPS	N/A	N/A	N	Serum	0.23	N
35	NPS	N/A	N/A	N	Serum	2.11	P
36	NPS	N/A	N/A	N	Serum	0.20	N
37	NPS	N/A	N/A	N	Serum	0.74	N
38	NPS	N/A	N/A	N	Serum	0.04	N
39	NPS	N/A	N/A	N	Serum	0.14	N
40	NPS	N/A	N/A	N	Serum	0.40	N
41	NPS	N/A	N/A	N	Serum	0.11	N
42	NPS	N/A	N/A	N	Serum	0.14	N
43	NPS	N/A	N/A	N	Serum	0.22	N
44	NPS	N/A	N/A	N	Serum	0.81	N
45	NPS	N/A	N/A	N	Serum	0.34	N

Number	Sample type	C_t value			Sample type	Peak current	
		RdRP	E	P/N		Result	P/N
46	NPS	N/A	N/A	N	Serum	0.00	N
47	NPS	N/A	N/A	N	Serum	0.00	N
48	NPS	N/A	N/A	N	Serum	0.00	N
49	NPS	N/A	N/A	N	Serum	0.23	N
50	NPS	N/A	N/A	N	Serum	0.00	N
51	NPS	N/A	N/A	N	Serum	0.88	N
52	NPS	N/A	N/A	N	Serum	0.00	N
53	NPS	N/A	N/A	N	Serum	1.09	N

Supplementary Table 5. Summary of the ROC curve analysis of the clinical samples for electrochemical detection. P values were calculated using two-sided Student's t-test.

	ORF1a gene	Nucleocapsid protein	IgG antibody
**Area under the curve**	1.00	0.996	0.993
**P value**	<0.0001	<0.0001	<0.0001
**Cut-off current values (μA)**	2.12	0.857	1.3
**Sensitivity**	1.00	0.98	0.97
**Specificity**	1.00	0.95	0.95
**Number of positive samples**	40	40	33
**Number of negative samples**	20	20	20

Supplementary Table 6. Comparison of our method with previous biosensing devices

Method	Target	LOD	Antifouling activities	Ref
Electrochemical sensor	IgG, IgM	10.1 ng mL^{-1} for IgG<br>1.64 ng mL^{-1} for IgM	-	[1]
Paper-based electrochemical sensor	IgG, IgM	0.96 ng mL^{-1} for IgG<br>0.14 ng mL^{-1} for IgM	-	[2]
Electrochemical sensor	S gene, Spike protein, Spike antibody	S gene: 10 fM<br>Spike protein: 30 pg mL^{-1}<br>Spike antibody: 100 pg mL^{-1}	-	[3]
OECT-based sensor	Acetic acid	10 µM	Consistency in transconductance in 10 mg/mL BSA solution	[4]
Electrochemical sensor	IgG	50 ng mL^{-1} for IgG	38% of streptavidin adsorption ratio (Bare gold: 86% of adsorption ratio)	[5]
Electrochemical sensor	kanamycin	-	Consistency in peak current over 2 hours in whole blood	[6]
Emulsion-based electrochemical biosensors<br>(Our device)	ORF1a gene, Nucleocapsid protein, IgG	ORF1a gene: 0.22 copies μL^{-1}<br>Nucleocapsid protein: 1.9 pg mL^{-1}<br>IgG: 20.4 pg mL^{-1}	Consistency in peak current over 1 month (retention of 101.01% in 1% BSA, 98.25% in NPS, 102.67% in serum)	This work

Supplementary references

1. Peng, R., et al. SPEEDS: A portable serological testing platform for rapid electrochemical detection of SARS-CoV-2 antibodies. Biosens. Bioelectron. 197, 113762 (2022)

2. Yakoh, A., et al. Paper-based electrochemical biosensor for diagnosing COVID-19: Detection of SARS-CoV-2 antibodies and antigen. Biosens. Bioelectron. 176, 112912 (2021)

3. Dou, Y., et al. A smartphone-based three-in-one biosensor for co-detection of SARS-CoV-2 viral RNA, antigen and antibody. Chem. Commun. 58(41), 6108 (2022)

4. Feng, J., et al. All-Polymer Fiber Organic Electrochemical Transistor for Chronic Chemical Detection in the Brain. Adv. Funct. Mat. 33, 2214945 (2023)

5. Kilic, T., et al. Zwitterionic polymer electroplating facilitates the preparation of electrode surfaces for biosensing. Adv. Mater. 34 (8), 2107892 (2022)

6. Chan, D., et al. Combinatorial polyacrylamide hydrogels for preventing biofouling on implantable biosensors. Adv. Mater. 34 (24), 2109764 (2022)
