# Supplementary material for: Micrometer-thick and porous nanocomposite coating for electrochemical sensors with exceptional antifouling and electroconducting properties
Source: Nat Commun. 2024 Feb 8;15:711. doi: 10.1038/s41467-024-44822-1 (PMC10853525; doi:10.1038/s41467-024-44822-1)
Supplement: Supplementary file 2 — Reporting Summary [file 41467_2024_44822_MOESM2_ESM.pdf]

nature portfolio

Corresponding author(s): Donald E. Ingber

Last updated by author(s): Jan 3, 2024

Reporting Summary

Nature Portfolio wishes to improve the reproducibility of the work that we publish. This form provides structure for consistency and transparency in reporting. For further information on Nature Portfolio policies, see our Editorial Policies and the Editorial Policy Checklist.

Statistics

For all statistical analyses, confirm that the following items are present in the figure legend, table legend, main text, or Methods section.

n/a	Confirmed	
☐	☒	The exact sample size (*n*) for each experimental group/condition, given as a discrete number and unit of measurement
☐	☒	A statement on whether measurements were taken from distinct samples or whether the same sample was measured repeatedly
☐	☒	The statistical test(s) used AND whether they are one- or two-sided<br>*Only common tests should be described solely by name; describe more complex techniques in the Methods section.*
☒	☐	A description of all covariates tested
☒	☐	A description of any assumptions or corrections, such as tests of normality and adjustment for multiple comparisons
☐	☒	A full description of the statistical parameters including central tendency (e.g. means) or other basic estimates (e.g. regression coefficient) AND variation (e.g. standard deviation) or associated estimates of uncertainty (e.g. confidence intervals)
☐	☒	For null hypothesis testing, the test statistic (e.g. *F*, *t*, *r*) with confidence intervals, effect sizes, degrees of freedom and *P* value noted<br>*Give P values as exact values whenever suitable.*
☒	☐	For Bayesian analysis, information on the choice of priors and Markov chain Monte Carlo settings
☒	☐	For hierarchical and complex designs, identification of the appropriate level for tests and full reporting of outcomes
☐	☒	Estimates of effect sizes (e.g. Cohen's *d*, Pearson's *r*), indicating how they were calculated

Our web collection on statistics for biologists contains articles on many of the points above.

Software and code

Policy information about availability of computer code

Data collection	Potentiostat measurement; ZIVE SP1, WonATech, the oxidation current was calculated using IVMAN 1.5 software.
Data analysis	Peak oxidation current for electrochemical assay was calculated using IVMAN 1.5 software. All data were plotted, and statistical tests were performed using Origin. ROC curve analysis was done in Origin. The figures were made using Powerpoint or Illustrator.

For manuscripts utilizing custom algorithms or software that are central to the research but not yet described in published literature, software must be made available to editors and reviewers. We strongly encourage code deposition in a community repository (e.g. GitHub). See the Nature Portfolio guidelines for submitting code & software for further information.

Data

Policy information about availability of data

All manuscripts must include a data availability statement. This statement should provide the following information, where applicable:

- Accession codes, unique identifiers, or web links for publicly available datasets
- A description of any restrictions on data availability
- For clinical datasets or third party data, please ensure that the statement adheres to our policy

All data needed to evaluate the findings can be found in the paper and its Supplementary Information. Source data for the figures and supplementary information are provided as a Source data with this paper.

Research involving human participants, their data, or biological material

Policy information about studies with human participants or human data. See also policy information about sex, gender (identity/presentation), and sexual orientation and race, ethnicity and racism.

Reporting on sex and gender	All human samples used in this study had been de-identified.
Reporting on race, ethnicity, or other socially relevant groupings	Not Applicable. There are no socially constructed or socially relevant categorization variables in this study.
Population characteristics	The nasopharyngeal specimens were collected from actively infectious patients, and the clinical serum samples were collected from convalescent patients.
Recruitment	De-identified clinical nasopharyngeal specimens and clinical serum samples from Gyeongsang National University College of Medicine (Korea, Republic of) were obtained from under their ethical approvals.
Ethics oversight	The protocol was reviewed and approved by the Institutional Review Board of Gyeongsang National University College of Medicine in Changwon, Korea (IRB approval number: 2022-10-012). Informed consent was obtained from all participants for research use.

Note that full information on the approval of the study protocol must also be provided in the manuscript.

Field-specific reporting

Please select the one below that is the best fit for your research. If you are not sure, read the appropriate sections before making your selection.

☒ Life sciences ☐ Behavioural & social sciences ☐ Ecological, evolutionary & environmental sciences

For a reference copy of the document with all sections, see nature.com/documents/nr-reporting-summary-flat.pdf

Life sciences study design

All studies must disclose on these points even when the disclosure is negative.

Sample size	The SARS-CoV-2 ORF1a gene, nucleocapsid protein, and IgG antibody were tested in 60, 60, and 53 clinical samples, respectively. Sample sizes were three biological replicates, which is a standard sample size in the field.
Data exclusions	No acquired data were excluded from the analyses.<br><br>The electrochemical data could be excluded during acquisition if voltammograms or impedance spectra showed only noise, clearly indicating that the electrodes or electric paths were defective (cut) or that connections were not proper. Electrochemical data could also be eliminated during acquisition owing to human mistake, for instance starting the experiment in the wrong conditions, or owing to accidents during acquisition (for example, touching the electrodes during measurement or spilling the solution). All these issues were always confirmed before repeating the experiment.
Replication	Electrochemical data described in biological samples were obtained from three technical replicates.
Randomization	Positive and negative clinical samples for the electrochemical detection of the SARS-CoV-2 ORF1a gene, nucleocapsid protein, and IgG antibody were randomly selected.
Blinding	The investigators were blinded to group allocation during data acquisition and analysis.

Reporting for specific materials, systems and methods

We require information from authors about some types of materials, experimental systems and methods used in many studies. Here, indicate whether each material, system or method listed is relevant to your study. If you are not sure if a list item applies to your research, read the appropriate section before selecting a response.

Materials & experimental systems

n/a	Involved in the study	
☐	☒	Antibodies
☒	☐	Eukaryotic cell lines
☒	☐	Palaeontology and archaeology
☒	☐	Animals and other organisms
☒	☐	Clinical data
☒	☐	Dual use research of concern
☒	☐	Plants

Methods

n/a	Involved in the study	
☒	☐	ChIP-seq
☒	☐	Flow cytometry
☒	☐	MRI-based neuroimaging

Antibodies

Antibodies used	FITC-labeled anti-IgG (Sigma-Aldrich, USA, no. F9512), SARS-CoV-2 nucleocapsid polyclonal antibody (Invitrogen, PA1-41386), biotinylated Anti-SARS-CoV-2 nucleocapsid protein antibody (Abcam, ab284656), biotin-SP AffiniPure F(ab')$_2$ Fragment Goat Anti-Human IgG (Jackson ImmunoResearch, 109-066-170), IgG (Invitrogen, MA5-35939)
Validation	FITC-labeled anti-IgG (Sigma-Aldrich, USA, no. F9512) was validated by immunofluorescence imaging (https://www.sigmaaldrich.com/US/en/product/sigma/f9512).<br>SARS-CoV-2 nucleocapsid polyclonal antibody (Invitrogen, PA1-41386) was validated by immunoblotting and tested on a cell line transfected with full-length SARS Nucleocapsid cDNA with a predicted molecular weight of 46 kDa (https://www.thermofisher.com/antibody/product/SARS-Coronavirus-Nucleocapsid-Antibody-Polyclonal/PA1-41386).<br>Biotinylated Anti-SARS-CoV-2 nucleocapsid protein antibody (Abcam, ab284656) was validated by ELISA (https://www.abcam.com/products/primary-antibodies/biotin-sars-cov-2-nucleocapsid-protein-antibody-85c10-ab284656.html).<br>Biotin-SP AffiniPure F(ab')$_2$ Fragment Goat Anti-Human IgG (Jackson ImmunoResearch, 109-066-170) was validated by immunoelectrophoresis and ELISA (https://www.jacksonimmuno.com/catalog/products/109-066-170).<br>IgG (Invitrogen, MA5-35939) was validated by ELISA (https://www.thermofisher.com/antibody/product/SARS-CoV-2-Spike-Protein-S1-Chimeric-Antibody-clone-H6-Recombinant-Monoclonal/MA5-35939).

Plants

Seed stocks	N/A
Novel plant genotypes	N/A
Authentication	N/A
